# JC Virus in Kidney Transplant Population: Are We Cautious Enough?

**DOI:** 10.3390/jcm13082217

**Published:** 2024-04-11

**Authors:** Mirha Pjanic, Mirna Aleckovic-Halilovic, Nikolina Basic-Jukic

**Affiliations:** 1 Clinic for Internal Diseases, Department of Nephrology, Dialysis and Kidney Transplantation, University Clinical Center Tuzla, 75000 Tuzla, Bosnia and Herzegovina; mirna.aleckovic@yahoo.com; 2 Department of Nephrology, Arterial Hypertension, Dialysis and Transplantation, University Hospital Centre Zagreb, 10000 Zagreb, Croatia; nikolina.basic.jukic@kbc-zagreb.hr

**Keywords:** JC virus, polyomavirus nephropathy, progressive multifocal leucoencephalopathy, kidney transplantation

## Abstract

The John Cunningham virus (JCV) is a polyomavirus that usually infects people at a young age and does not cause any symptoms in immunocompetent individuals. However, in immunocompromised individuals, such as kidney transplant recipients, JCV can cause severe and potentially fatal disease. Unfortunately, JCV has not been researched as extensively as the BK virus and is not mentioned in relevant kidney transplant guidelines. This lack of attention to JCV can lead to less consideration in kidney transplant patients’ care. Surveillance using locally available diagnostic methods is of the utmost importance. The presence of JCV can be diagnosed with urine decoy cells, viruria, or viremia verified by the PCR method. A low threshold for considering JCV as a possible cause of any neurological or renal dysfunction in kidney transplant recipients must be maintained. In such cases, kidney and brain biopsy are indicated. Maintaining the appropriate immunosuppression while avoiding over-immunosuppression to prevent JCV disease is crucial, and the approach should be individual, according to overall immunological risk. We hypothesize that the presence of the JCV can indicate overt immunosuppression and identify kidney transplant recipients more prone to opportunistic infections and diseases, including some malignancies. To explore that, future observational studies are needed.

## 1. Introduction

The John Cunningham virus (JCV) is a human polyomavirus that was first discovered in 1971 in an immunodeficient patient with Hodgkin’s disease, causing progressive multifocal leukoencephalopathy (PML), a condition that is best known for [1]. Primary infection with JCV typically happens at a young age via the fecal–oral or respiratory route, and it is usually asymptomatic or oligosymptomatic in immunocompetent individuals [2]. After that, the virus stays latent in the kidney, central nervous system, tonsillar cells, and hematopoietic progenitor cells without causing symptoms, thanks to the competent immune system, especially JC virus-specific T lymphocytes [2]. However, immunological changes, such as initiating immunosuppressive therapy, can break the balance between the virus and the host, and thus result in disease [2].

Different patterns of reactivation of JCV and the other virus from the same family of Polyomavirinae, the BK virus (BKV), after kidney transplantation are described in the literature [3]. In 2010, Sanudh et al. performed a study among 30 kidney transplant recipients using in-house, quantitative, real-time, multiplex polymerase chain reaction (PCR). They found that in their cohort, JCV in urine occurred much earlier, at a median of 5 days after the transplantation, compared to BK viruria, which was seen after 3–6 months [3]. However, according to our literature search, no study on a larger study population confirms those findings or demonstrates different manners of reactivation.

Although they belong to the same family and do not differ in the severity of the pathology they cause, BKV is far more researched and represented in the literature than JCV, leading to less consideration of JCV in kidney transplant patients’ care. This is supported by the fact that there is no mention of JCV in the relevant kidney transplant guidelines [4,5,6,7,8], which motivated us to write this article. We reviewed the literature specific to data on JCV in kidney transplant patients, regarding the screening, clinical presentation, diagnosis, management, and prevention of JCV infection and/or JCV disease. Particular interest was given to analyzing the clinical significance of asymptomatic JCV infections in indicating the inappropriate level of immunosuppression leading to undesirable immunological events and infections.

## 2. The First Step Is the Hardest—Clinical Suspicion of JCV Disease

Although all immunocompromised patients are susceptible to JCV disease, kidney transplant patients are particularly at risk due to factors such as human leukocyte antigen (HLA) mismatch between donor and recipient, long periods of cold ischemia, continual efforts by the host to reject the transplanted kidney, and the toxic effects of immunosuppressive drugs. These factors, when combined, lead to persistent injury to the transplanted kidney, thus creating a perfect milieu for the thriving of the virus and causing the disease [9]. PML is the deadliest condition that JCV can cause. Even though only 15 cases of PML in kidney transplant recipients were described in the literature, a high index of suspicion must be maintained in kidney transplant patients with any neurological symptoms [10,11]. The typical symptoms are visual and cognitive impairment, hemiparesis, seizures, ataxia, and cranial nerve disorders [11]. However, Bialasiewicz et al. reported a case of an unusual presentation of PML caused by JCV in a kidney transplant recipient who was JCV seronegative before the transplantation, in contrast to the seropositive donor. The presentation included thrombosis of the cerebral venous sinus and the absence of characteristic radiological lesions. Due to an atypical presentation, the patient’s diagnosis was delayed and was ultimately made with PCR analysis of the cerebrospinal fluid (CSF). The patient required cessation of immunosuppression therapy and graft nephrectomy to survive [11]. As for the rest of the cases mentioned above of PML in kidney transplant patients, 9 of the 14 patients died [10]. In all five of the patients who survived, immunosuppressive therapy was reduced or altogether ceased, except for methylprednisolone in some cases. Of those five patients, immunosuppressive therapy was discontinued in two patients, chronic dialysis was started, and graft nephrectomy was performed in one. Two patients recovered with a functional kidney graft. One patient also recovered with a functioning kidney graft but died 14 months later from sepsis. Unfortunately, only six patients were alive at the time of the diagnosis of PML. Out of the six, four patients were diagnosed with PCR of the CSF, and two patients were diagnosed with a brain biopsy. The remaining eight patients were diagnosed with PML after their death at autopsy [10].

As for JCV nephropathy, it can occur early or late after transplant, unlike BKV nephropathy, which usually occurs early after kidney transplantation [12]. No characteristic features would immediately direct the clinician to a possible diagnosis of JCV nephropathy. Rao et al. reported a case series of three asymptomatic patients with JC viremia and viruria detected by protocolar screening in the early post-transplant period [13]. A kidney biopsy was not performed because no renal dysfunction was present. All the patients underwent an alteration of and a reduction in their immunosuppressive maintenance therapy. Although the viremia disappeared within five months, the viruria persisted, and the patients remained asymptomatic [13]. However, there are some more severe cases described in the literature. Aguilar et al. reported a case of a heart and kidney transplant patient whose kidney function worsened seven years post-transplant with bland urinary sediment, and diagnosis of polyomavirus nephropathy was made based on kidney biopsy result that demonstrated positive immunohistochemical staining for SV40 polyomavirus in the tubular epithelial cells and tubulointerstitial inflammation [14]. Electronic microscopy confirmed the presence of polyomavirus particles in the paracrystalline array arrangement. However, the diagnosis of JCV nephropathy was only confirmed after ruling out BKV. Subsequently, the patient’s urine and serum PCR samples came back positive for JCV. The patient’s kidney function stabilized despite JC viremia variation after stopping mycophenolate mofetil (MMF) and multiple intravenous immunoglobulins (IVIG) courses [14]. Rasonable et al. investigated 263 solid organ transplant recipients and found JCV in blood in 2.7% of kidney transplant recipients [15]. There were no cases of PML among the study population, and most patients were asymptomatic [15]. Drachenberg et al. demonstrated in their cohort of kidney transplant recipients that JCV viruria, compared to BKV viruria, was more asymptomatic and more present in older patients [16]. Significant biopsy findings in patients with JCV nephropathy were inflammation and fibrosis, but JCV nephropathy was less common than that of BKV, with incidences of 0.9% and 5.5%, respectively. Only 14.2% of patients had JCV viremia, described as transient and low. More than half of the patients with JCV continued to shed decoy cells despite the reduction in immunosuppressive therapy, but there were no cases of graft loss among those patients [16].

Lopez et al. investigated the prevalence of both JCV and BKV in their cohort of 186 kidney transplant recipients [17]. Approximately one-third of the study population had polyomavirus viruria, which JCV and BKV equally caused. None of the patients with JC viruria developed viremia and JCV nephropathy, unlike BKV-positive patients [17]. Wiegley et al. searched the pathology archives in two centers and found seven cases of JCV nephropathy described. All biopsies, except one, demonstrated the same findings as mentioned—foci of interstitial fibrosis with nonspecific inflammation. The exception was one biopsy result from a patient who presented earlier than others and had active inflammation and tubulitis [12]. Two biopsies were from patients who presented after being treated for acute graft rejection. Cytopathic changes correlated with the histological viral load and were not present in cases with low viral load, but staining for SV40 was positive [12]. Unfortunately, data on the viruria and viremia of these patients were unavailable to us. Still, it is important to mention that the authors concluded that the absence of BK viruria and/or viremia can be deceitful [12].

There have been anecdotal reports suggesting that JCV can cause other clinical diseases beyond PML and JCV nephropathy [18,19]. For instance, there are documented cases in the literature where immunocompromised patients, such as liver and bone marrow transplant patients, experienced peripheral neuropathy as the first clinical sign of PML caused by JCV [18,19]. Mohanty et al. confirmed the diagnosis of peripheral neuropathy through a biopsy that demonstrated segmental demyelination as a sign of JCV effects outside of the central nervous system [18]. Also, a systematic review and meta-analysis on the association of JCV and colorectal cancer performed by Shavaleh et al. showed a JCV prevalence of 43% in colorectal cancer tissues [20]. The authors demonstrated that the presence of JCV in colorectal tissues increased the likelihood of colorectal cancer by 4.70 times. The potential role of JCV in colorectal cancer could be explained by the large T antigen (T Ag) of the JCV interfering with the normal cell cycle, resulting in genetic instability and oncogenic effect [20].

We conclude that in vulnerable kidney transplant patients, it is crucial to thoroughly assess any signs or symptoms of neurological or kidney dysfunction. The evaluation should include testing for viral opportunistic infections, such as JCV, as they can quickly escalate and result in a negative outcome for both the patient and the graft. Therefore, prompt and comprehensive evaluation and timely diagnosis are essential for a successful outcome. Also, this association of JCV and colorectal carcinoma reminds us of the importance of screening for colorectal carcinoma in kidney transplant recipients, especially those with JCV—despite some being asymptomatic—as well as in the general population.

## 3. Diagnostic Approaches

In the previously mentioned study by Drachember et al. the initial test for JCV screening in their cohort of kidney transplant recipients was urine cytology for decoy cells [16]. If any decoy cell was present, the result was considered positive; in their study, it was 13.8%. As for the dynamics of urine cytology testing, it was performed according to the protocol as follows: once monthly until three months post-transplant, then every three months until the end of the study, or when serum creatinine rose at least 20% from baseline. All positive urine samples for the decoy cells were then tested by PCR for JCV and BKV, as well as serum samples from those patients. JCV viruria was found in 27.2% of patients, and 16.5% of patients with present decoy cells had positive PCR of the urine for both JCV and BKV. Among patients with JCV viruria, six patients (21.4%) had features of polyomavirus nephropathy in their biopsy results. Of those six patients, four also had JCV viremia. Renal biopsy samples (one or more sections, if the first section was negative or inconclusive) were stained for SV40 large T antigen cross-reacting with JCV and BKV. If staining for SW40 was positive in renal epithelial cells, polyomavirus nephropathy was diagnosed [16]. There are three histologic patterns of polyomavirus nephropathy, as follows: A—present cytopathic changes but scarce or nonexistent inflammation or tubular atrophy, B—present inflammation and partial tubular atrophy with viral cytopathic changes, C—chronic inflammation, diffuse tubular atrophy and fibrosis, and scarce viral cytopathic changes [16]. As for histological findings in patients with JCV nephropathy, they were uniform and had no significant differences compared to histologic changes in BKV nephropathy. Overall, cytopathic changes were scarce, and it is important to note that in JCV nephropathy, compared to BKV nephropathy, significantly fewer infected cells were found. Other observed features were nonspecific and included chronic inflammation and fibrosis. In patients with JCV decoy cell shedding, there were no cases of graft dysfunction, in contrast to findings of graft dysfunction in 38% of patients with BKV shedding. JCV decoy cell shedding was present at the end of the 3-year study period in approximately half of the patients (57.1%) but with no graft loss. All patients with JCV nephropathy had stable serum creatinine at the time of the biopsy. A total of 6 of the 28 patients with JCV decoy cell shedding had an increase in serum creatinine at the time of the biopsy. However, biopsy findings pointed in the direction of other causes of graft dysfunction, and a control renal biopsy was performed in four of these patients six months after the initial biopsy and continuous JCV shedding, confirming the absence of polyomavirus nephropathy or acute rejection. In 14.3% of the patients shedding JCV decoy cells, concurrent viremia was present. Still, it did not last for over a month, with significantly fewer viral copies than patients with BKV shedding [16].

In resourceful centers that can afford it, positive SW40 staining can be supplemented with JCV-specific DNA in situ hybridization (ISH) staining in paraffin-embedded renal biopsy tissue, as was done in the case described by Abu Jawdeh and colleagues [21].

As for the diagnosis of PML, diagnosis is based on the characteristic MRI findings of multifocal demyelinating patches located anywhere in the central nervous system, and positive JCV PCR in CSF with a sensitivity of 58% and specificity of 92–100% [22]. The gold standard is the brain biopsy, with a sensitivity of 64–96% and a specificity of 100%, demonstrating demyelinating lesions, gliosis, and abnormal astrocytes and macrophages [22]. PCR of the brain tissue can also be performed [22].

In summary, performing any form of surveillance is better than none. Whether due to the decoy cells in the urine, viruria, or viremia verified by the PCR method, these are all adequate signals to decrease immunosuppression, considering the overall individual immunological risk. If there is elevated creatinine, newly occurring microalbuminuria or its increase from baseline values, or growing viral load, we recommended doing a kidney biopsy. If there are no advanced confirmatory methods available, and there are clinical changes as described above and/or significant histologic changes without any other causes, then the diagnosis of PVN can be made. Additionally, it is important to keep a low detection threshold for any neurological symptoms.

## 4. Interactions with Other Viruses and Their Clinical Implications

Chen et al. provided evidence of a negative interaction between JCV and BKV in kidney transplant patients [23]. They performed a study on approximately 200 kidney transplant patients who were initially screened for BKV. They found JCV viruria in 16% of patients; in all cases, it was in the early post-transplant period. None of the patients with JCV viruria developed JCV viremia or PML. Compared to patients with BKV viruria, patients with JCV viruria were older. The authors emphasized another difference between JCV and BKV, based on comparing kidney recipients from the same donor—BKV most likely reactivates in a kidney graft, in contrast with the JCV that reactivates in the native kidney, following kidney transplantation and immunosuppression initiation. There was no difference in graft survival between patients with JCV viruria and those without JCV viruria. However, the rate of acute rejection was higher in patients without JCV viruria compared to those with JCV viruria, both in the first year (7% vs. 0%, respectively) and in 5 years (14% vs. 7%, respectively). The authors explained it by observing the presence of JCV viruria as a surrogate for adequate immunosuppression. As for the concurrence of JCV and BKV, JCV viruria was significantly more present in kidney transplant patients without BKV viruria compared to recipients with BKV viruria (22% vs. 4%, respectively). Further on, JCV viruria was inversely associated with BKV seropositivity of both donor and recipient. The coactivation rate among their study population was only 1.6%, which also points to the negative association between JCV and BKV. Possible mechanisms are the direct inhibition or competition for the same cellular sites for replication, and overall humoral immune response against both viruses. In that manner, the donor’s BKV antibodies, which were positively associated with the recipient’s BK viruria, were negatively associated with JCV viruria. A negative relationship was also observed between the recipient BKV antibody titer and JCV viruria. The study population had no JCV viremia or nephropathy [23]. Saundh et al. also demonstrated in their cohort of kidney transplant recipients that recipients from the JCV-negative donors were twice as likely to develop a BKV infection [24].

It is expected that JCV can interact with viruses other than polyomaviruses. Some interesting cases in the literature describe patients with JCV disease and a preceding Cytomegalovirus (CMV) infection [18,19]. In two cases where JCV-positive patients experienced peripheral neuropathy as the initial manifestation of PML, it followed an episode of CMV infection [18,19]. Owen et al. reported a case of a bone marrow transplant patient who developed a CMV disease [25]. The patient manifested with neutropenia and lymphocytosis consisting of large granular lymphocytes and developed PML a year later. The outcome was fatal [25]. It is doubtful that these events are coincidental, given the fact that the stimulating effect of the CMV on JVC’s replication is also found under in vitro conditions [26]. Certain viral infections can have a significant impact on the health of kidney transplant patients. CMV’s immunomodulatory effects, especially after kidney transplantation, are well known; it can additionally weaken the immune system and increase the risk for other viral infections, such as JCV.

In the post-COVID era, we are still learning about the long-term consequences of the COVID-19 disease. However, cases of COVID-19 have already been described as a predisposing factor in the occurrence of other viral infections, such as the Respiratory syncytial virus (RSV) and Influenza virus [27,28]. Therefore, the already described cases of JCV infection preceded by COVID-19 disease in kidney transplant recipients are not surprising [21]. Abu Jawdeh et al. described a patient with JCV nephropathy late after simultaneous heart and kidney transplantation, which was preceded by the COVID-19 disease [21]. However, the authors rightly consider that not only the COVID-19 infection but also the therapy the patient received for it (convalescent plasma and tocilizumab) possibly modulated the immune system, making the patient prone to JCV disease [21].

It should be emphasized that there are also non-immunological risk factors for developing JCV disease. Keykhosravi et al. found that diabetes mellitus and kidney stones were significantly associated with JCV after kidney transplantation and urinary reflux [29].

Monitoring the Torque Teno Virus (TTV) DNA load in kidney transplant recipients’ blood is one of the indirect and non-invasive methods that can indicate overimmunosuppression. However, this method is only available in some resourceful transplant centers [30].

Therefore, it is crucial to take a holistic approach when treating transplant patients and not view viral infections as isolated events. Instead, we should consider their implications for overall patient health and kidney graft survival.

## 5. Therapeutic Options

Reduction in immunosuppressive therapy remains the cornerstone of treatment of JCV disease. Some authors interpret JCV infection as a sign of excessive immunosuppression [26]. Based on the literature data, the initial therapeutic steps typically involve reducing the tacrolimus dose (trough levels between 3–5 ng/mL) and mycophenolate mofetil dose (MMF) [10,11,14]. Some authors also suggest switching from MMF to azathioprine, given the similar long-term outcomes of MMF and azathioprine in kidney transplant patients [31,32]. Conversion from tacrolimus to sirolimus is also a possible therapeutic option, according to case reports described in the literature [33]. Intravenous immunoglobulins (IVIG), given their broad immunomodulatory effects, are used in the therapy of almost all symptomatic, moderate, and severe cases [10,11,14,33]. However, it is important to note the challenge of providing sufficient dosage, especially in resource-limited countries where only limited amounts of IVIG are available. In life-threatening cases, gradual but accelerated cessation of immunosuppressive therapy and graft nephrectomy are necessary to save the patient’s life [10,11]. There have been reports on the effectiveness of certain drugs for treating JCV disease in immunocompromised patients with or without nephropathy [34,35,36,37,38]. These drugs include mirtazapine, cytarabine, leflunomide, cidofovir, quinolones, and sorafenib. However, most of the reports are anecdotal and mostly about patients with AIDS and PML. None of the reports demonstrated definitive clinical benefit of the above-mentioned drugs.

Although there is significantly less research compared to research on BKV, there is still some research into new therapeutic options for severe cases of JCV, albeit few. Based on the previous reports on using JCV or BKV-specific T cells from allogeneic donors in the treatment of PML, Cortese et al. performed a pilot study to evaluate the efficacy of treatment with partially HLA-matched donor-derived BKV-specific T cells (PyVST) for patients with PML [39,40,41,42]. The study was open label, following one cohort without a control group [42]. Donor candidates were first-degree relatives without contraindications for leukapheresis, with at least two HLA-matching alleles required, and JCV seropositive donors were preferable. The study enrolled 12 patients with poor prognoses who had previously been treated for PML but with a progression of the disease. The most common underlying condition was lymphoproliferative disease. Any treatment that could be immunomodulatory or interfere with the PyVST was not permitted. Patients received one initial dose and up to two subsequent doses of PyVST. All 12 patients received at least one infusion; 10 received a second, and 7 a third. Seven patients survived PML for at least one year from PyVST treatment initiation. Five patients died of PML in the period of approximately two months after receiving the last dose of PyVST. Their substantial clinical deterioration with concurrent MRI lesions worsening led to withdrawal from the study [40]. It would be interesting to compare the JCV burden before and after the treatment with PyVST, for which we must wait for future studies. Recently, Kaiserman et al. provided a comprehensive review of all the drugs that have shown possible clinical benefits by inhibiting the infection and spread of the JCV, but only for patients with PML [43]. They also presented a novel compound (GW-5074) that showed promising antiviral activity in vitro by inhibiting the signal transmission process in virus-infected cells. However, they failed to demonstrate a similar effect in vivo. The authors concluded that further investigation of molecules and compounds with direct antiviral effects on JCV is necessary [43]. However, being still far away from that kind of therapy, and considering the limited benefits of all the drugs mentioned above and that a definitive cure for JCV disease is still not available, we conclude that it is better to focus on surveillance and prevention as the saying goes, ‘An ounce of prevention is worth a pound of cure’.

## 6. The Essential Role of Surveillance

Although the guidelines for kidney transplant patients do not provide recommendations for JCV, all articles addressing this issue emphasize the same—we must put more effort into screening and identifying the risk factors for JCV disease. Surveillance activities should start prior to the transplantation and should include not just the kidney recipient but the donor as well. Both donor and recipient should undergo surveillance for JCV based on available diagnostic methods. After the transplantation, a trend in the number of virus copies in urine or blood, or decoy cells in urine, is needed to adequately monitor the recipient, as more than a single measurement is required. More rigorous surveillance after transplantation is required in recipients with higher immunosuppression burden and recipients with comorbidities that additionally compromise the immune system that is already weakened by uremia and immunosuppressive drugs. Protocol kidney biopsies also have an important role in the early detection of JCV nephropathy. However, most transplant centers, except university and research hospitals and institutes, do not perform protocolar biopsies routinely for all patients but only for high-risk patients [44,45].

For prevention, an individual approach should be taken with initial adjustment of immunosuppression for patients with low immunological risk and high risk of infection, and vice versa.

Suspecting JCV disease in the case of kidney dysfunction and/or neurological dysfunction in transplant patients is also of the utmost importance for a timely diagnosis and to avoid possible scenarios of the disease worsening to the point where the kidney or the patient cannot be saved.


**Our Recommendations for JC Virus Surveillance**
The JC virus can occur at any time after transplantation, unlike the BK virus, which most often occurs early after the transplantation, when the immunosuppression is the most intense. Therefore, we recommend that in the first two years after the transplantation, the JC virus is part of the surveillance with the BK virus, with dynamics by which surveillance for the BK virus is done. Besides surveillance, testing should also be done in case of a decline in kidney function or any neurological symptoms in kidney transplant patients. This approach should be considered as standard care.Two years after the kidney transplantation, we suggest surveillance according to the transplant center’s experience with the JC virus; we suggest it at least once every six months when there is unclear kidney insufficiency or neurological symptoms, or as indicated based on the patient’s total immunosuppressive load, for example, patients after re-transplantation and patients who had received additional immunosuppressive therapy due to graft rejection.When it comes to selecting a method for surveillance testing, it is better to have some testing than none. This means that any testing method that a transplant center can afford is acceptable. If there is a concern that a specific organ may be affected, then conducting a PCR test on a biopsy or puncture sample from the affected organ is considered to be the gold standard.In cases where transplant centers have limited resources, it is important to remember that the JC virus should not be overlooked as a potential cause of graft dysfunction and/or neurological symptoms in kidney transplant patients. Diagnosis should be confirmed through exclusion or, if necessary, by sending a sample for analysis to a larger center where testing for JC virus can be performed.In patients with detected JC virus, even if asymptomatic, screening for colorectal cancer (CRC) should not be overlooked, the dynamics of which should be more frequent than what is recommended for the general population, especially if other risk factors are present, e.g., positive family history for CRC.

## 7. Conclusions

Maintaining appropriate concentrations of immunosuppressive drugs while avoiding over-immunosuppression is essential to prevent JCV infection. If an infection occurs, it is important to consider JCV as a possible cause in diagnosing any neurological or renal dysfunction in a kidney transplant recipient. Prompt treatment is required to prevent the escalation of the disease, which can be fatal. To accomplish this, a dedicated holistic multidisciplinary approach is necessary.

## 8. Future Directions

There is a need for an observational study to explore different JCV clinical implications, including its predictive value as an indicator of achieved individual level of immunosuppression, and as a possible risk or predisposing factor for certain infections and malignancies. In that setting, we hypothesize that the presence of the JC virus in the urine or blood, although sometimes asymptomatic, could be used as an indicator of overt immunosuppression and, in that way, and/or through other more complex immunomodulatory (inter)actions, possibly identify the kidney transplant recipients that are more prone to opportunistic infections and diseases, and some malignant diseases.

## Data Availability

Not applicable.
